# Clinical utility of digital technologies in Parkinson’s disease

**DOI:** 10.1007/s00702-025-03062-3

**Published:** 2025-11-12

**Authors:** Björn Falkenburger, Alessandra Fanciulli, Monika Pötter-Nerger, Thilo van Eimeren, Joseph Classen, Günter Höglinger, Christoph Redecker, Jürgen Winkler, Jochen Klucken

**Affiliations:** 1 Department of Neurology, Technische Universität Dresden, Dresden, Germany; 2 Department of Neurology, Medical University of Innsbruck, Innsbruck, Austria; 3 Department of Neurology, University of Hamburg, Hamburg, Germany; 4 Department of Neurology, University Hospital Cologne, Cologne, Germany; 5 Department of Neurology, University of Leipzig, Leipzig, Germany; 6 Department of Neurology, German Center for Neurodegenerative Diseases (DZNE), LMU University Hospital, Ludwig-Maximilians-Universität (LMU) München, Munich, Germany; 7 Munich Cluster for Systems Neurology (SyNergy), Munich, Germany; 8 Department of Neurology, Medical School OWL, Klinikum Lippe, Bielefeld University, Lemgo, Germany; 9 Department of Neurology, University of Erlangen, Erlangen, Germany; 10 Luxembourg Centre for Systems Biomedicine (LCSB), Université du Luxembourg, Centre Hospitalier du Luxembourg, Esch-sur- Alzette, Strassen, Luxembourg, Luxembourg

## Abstract

Digital health technologies (DHTs) are on the verge of changing treatment of Parkinson’s disease (PD). This narrative overview article describes DHTs that are already used or in development, focusing on technologies available in Germany and on their clinical utility. Examples include applications that are primarily used by patients, i.e., tools for patient education and empowerment, but also stand-alone interventions improving clinical outcomes including motor and non-motor symptoms. In Germany, these patient-centered DHTs fall under the HTA framework of digital health applications (DIGA). DHT for telemonitoring, in contrast, are designed to support physicians and other healthcare providers and therefore medical devices. DHT are also used increasingly as exploratory endpoints in trials testing neuroprotective treatments.

## Introduction

Following the outbreak of the COVID-19 pandemic in 2020, many regular in-person healthcare visits were suspended to protect vulnerable populations and redirect resources to emergency settings. This situation further exacerbated the vulnerability of individuals with chronic neurological conditions such Parkinson Disease (PD), who depend upon continuous and multidisciplinary healthcare provision. Patients experienced increased isolation, difficulty accessing specialized care, and, in some cases, shortages of medical equipment and therapies instead. The pandemic thus placed considerable strain on traditional models of care, but it simultaneously boosted healthcare digitization in the form of telemedical consultations, telemonitoring of key health parameters, and remote interventions (Fanciulli et al., 2023).

For PD and other movement disorders, an increasing number of digital health technologies (DHTs) have been developed for objective assessment and/or remote monitoring of a variety of symptoms as reviewed by ourselves and others (Moreau et al. 2023; Ossig et al. 2016; Sapienza et al. 2024). Increasing numbers of clinical studies report on the precision and accuracy of digital tools such as wearable sensors to quantify digital mobility outcomes and related functional impairment (Mancini et al. 2025; Moreau et al. 2023). However, validation of their clinical utility, i.e., where the application of DHTs generates a benefit for patients and/or healthcare professionals, has so far received considerably less attention with only 9 in 296 publications addressing clinical value (Sapienza et al. 2024). During the COVID-19 pandemic, telerehabilitation indeed demonstrated feasible and beneficial effects on both parkinsonian motor and non-motor symptoms (Morris et al. 2021; Tamplin et al. 2021; Vellata et al. 2021). Such crisis-driven digitalization of healthcare provision stood the proof of time also beyond the pandemic horizon and holds potential for further abating costs, geographical barriers and resources constraints, ultimately contributing to a more equitable access to care.

Germany is the first healthcare system in Europe that provides the possibility to prescribe digital health applications (DIGA). The German DIGA belongs to a new class of “digital medical devices - DMDs” that are evidence-based validated DHTs (Boers et al., 2025; Lauer et al. 2021). They can serve as “diagnostic” or “therapeutic” components, but also inform or educate patients, or support healthcare management. Here, Germany has provided a health technology assessment (HTA) framework that systematically evaluates the clinical benefit of DHTs prior to reimbursement by the statutory health insurances, and thus provides access for innovative technologies to reach patients across all medical specialties (Arcà et al. 2025). DIGA are patient-facing applications designed to improve personal health and facilitate patient journeys through the healthcare system. For instance, DIGA can be prescribed with a given diagnose of depression, pain, fatigue and erectile dysfunction (Weimar et al. 2025). DIGA are patient-centered and have to provide a benefit directly for patients based on their digital functionality. They can include telemonitoring function, but the beneficiary of the evidence-based added value has to be the patient. Thus, the DIGA is distinct from other DHTs with telemonitoring function, that primarily support the healthcare professionals in e.g. remote symptom assessment and treatment monitoring. In addition, other medical devices may support the patient and/or the healthcare professional. Examples include Holter electrocardiograms, medication pumps and impulse generators for deep brain stimulation. For medical devices, a distinct regulatory framework exists by the EU (Medical Device Coordination Group 2019) – however, with a substantially lower requirement to the evidence level of the validation of medical benefits. Importantly, a broader acceptance by patients (Paccoud et al. 2024b) and healthcare professional, as well as regulator, policy maker and society is still missing (Paccoud et al. 2024a).

**Fig. 1:**
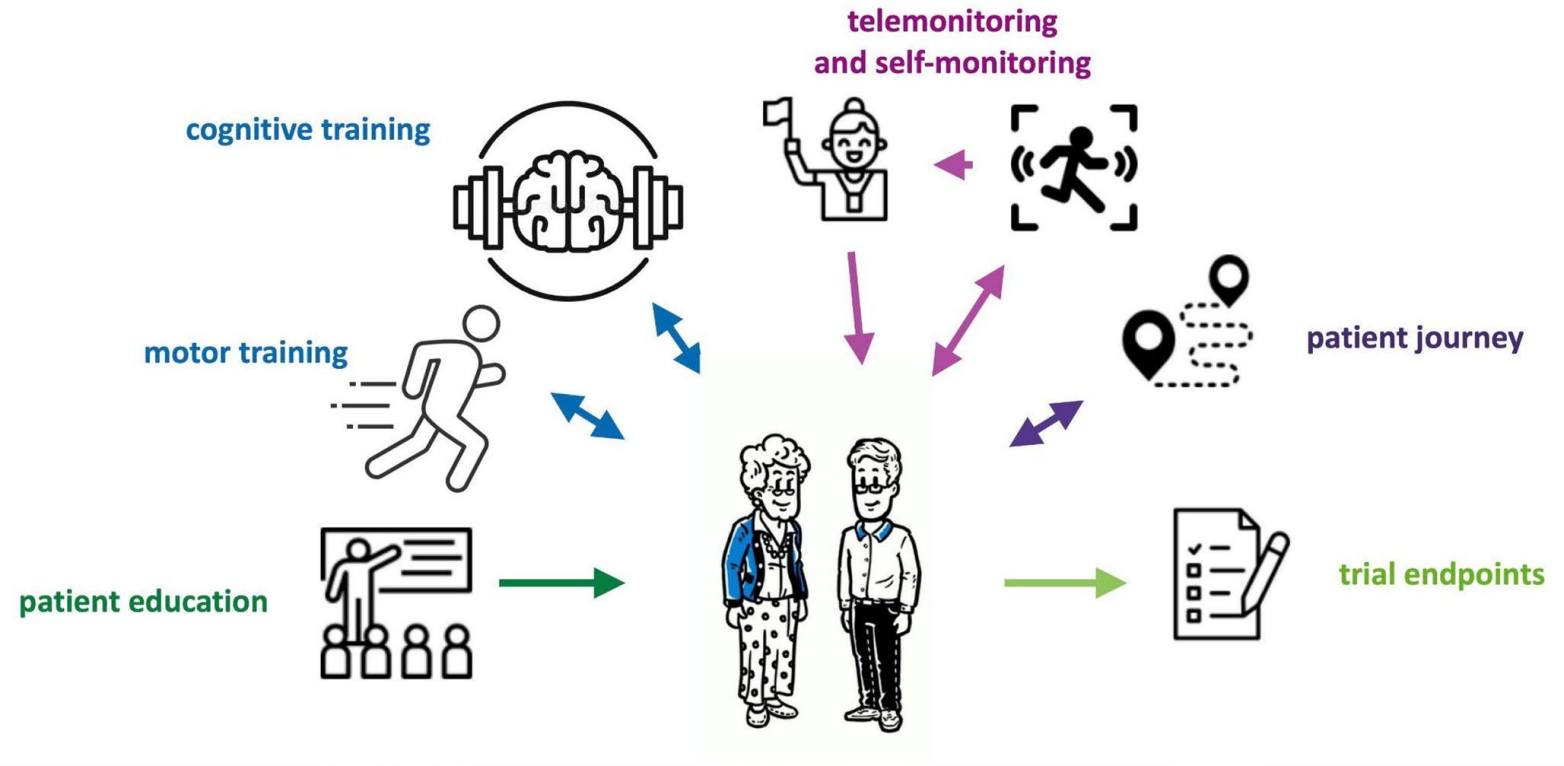
Summary of potential applications for DHTs in Parkinson’s disease

In this narrative overview article, we describe applications for which DHTs are currently used or developed in the context of PD, which include patient education, patient empowerment, telemonitoring and patient journey. Some applications are depicted in Fig. 1 and examples listed in Table 1. In this rapidly evolving field, information for many DHTs is only available from press releases and websites, but not yet published in scientific journals. The authors aimed for a comprehensive overview by selecting diverse examples that they know of. Still, this review is incomplete and biased for DHTs available in Germany. Clinical utility and research perspectives are discussed.

**Table 1:** Examples for digital technologies used in the context of Parkinson’s disease

Area of application	Examples for digital technologies (manufacturer, website)
Patient education and empowerment	Swiss Parkinson App (Parkinson Switzerland) https://www.parkinson.ch/selbsthilfe/parkinson-app Parkinson Journal App (Parkinson Journal) https://parkinson-journal.de/parkinson-journal-app/ Fox Wearable Companion app (Michel J. Fox Foundation) https://fox-insight-app.soft112.com/ PD buddy (Parkinson’s UK) https://pdbuddy.app/ My moves matter (Richelle Flanagan and Rene Reinbacher) https://www.mymovesmatter.com/ includes nutrition therapy through NutriPD Parkinson’s Disease Manager (part of the @Point of Care platform) StrivePD (iPhone, by Rune Labs)
Medication reminder	Mytherapy, Medisafe, Mediteo, Healthera, Apple Health
Gait problems, including freezing of gait	Dual-Task Gait and Balance Assessment (iPhone, described in: (Su et al. 2021) Rhythm – Parkinson’s Gait App (iPhone, by Andreas Ink) Walking Tall (by University of New South Wales) Foggy (iPhone, by h2o Therapeutics) Holocue (headset, by Vrije Universiteit Amsterdam, described in (Geerse et al. 2020, 2021) BIPBIP (Android, by Graziano Pravadelli) Cue2Walk (by Sander Minnoye and Martijn van der Ent) CuPiD (by University of Bologna, described in PMID: 26777408 (Ginis et al. 2016))
Depression (recently reviewed in PMID: 38260995 (Haaf et al. 2024))	deprexis (web application by GAIA AG, (Pearson et al. 2024) https://diga.bfarm.de/de/verzeichnis/00450 edupression (web application by SOFY GmbH) https://diga.bfarm.de/de/verzeichnis/01815 elona therapy Depression (app by Elona Health GmbH, (Atik et al. 2023) https://diga.bfarm.de/de/verzeichnis/01254 Novego (web application by IVPNetworks GmbH, (Beiwinkel et al. 2017) https://diga.bfarm.de/de/verzeichnis/01110 Selfapys (app by Selfapy GmbH, (Schefft et al. 2024) https://diga.bfarm.de/de/verzeichnis/00876 hello better (depression, sleep, pain, by GET.ON Institute, (Bernstein et al. 2024) https://hellobetter.de/
Other symptoms	Voice Trainer App (by Radboud University, Nijmegen, described in (Knuijt et al. 2024) NeuroNation (cognitive training, by Synaptikon GmbH, (Ferizaj et al. 2024) https://diga.bfarm.de/de/verzeichnis/01113 Kranus Edera (erectile dysfunction, by Kranus Health) https://diga.bfarm.de/de/verzeichnis/01282 elevida (fatigue, by GAIA AG, (Pöttgen et al. 2018) https://diga.bfarm.de/de/verzeichnis/00419 untire (fatigue, by Tired of Cancer B.V., (Spahrkäs et al. 2020) https://diga.bfarm.de/de/verzeichnis/01967 OrthoStat (orthostatic hypotension, app by Asterian)
Telemonitoring (systems used for treatment adjustments were recently reviewed in PMID 37919332 (Moreau et al. 2023))	PD-Monitor (by PD Neurotechnology) PKG (by Global Kinetics) STAT-ON (by Sense4care) DynaPort7 (by McRoberts) Kinesia 360 (by GreatLakes Neurotechnologies) Mobility Lab (by APDM Wearable Technologies) FeetMe Monitor Insoles (by FeetMe) ParkinsonGo (by Portabiles HealthCare Technologies)
Patient journey	“Der Parkinson-Lotse” (web portal, by Hilde-Ulrichs Foundation) http://aktive-parkinsonstiftung.de/parkinson-lotse JamesAktiv (web portal, by University of Münster) Doctolib (by Doctolib GmbH, Berlin)

## Patient information, education and empowerment

The substantial disease burden associated with chronic neurological conditions, such as PD, often prompts a completely understandable search for health information. Yet, access to helpful, reliable, and up-to-date resources remained limited until recently. Several websites collect and produce content for patient education, for instance websites of patient organizations and foundations (Table 1). Content includes articles, explanatory videos and scientific lectures. Several smartphone apps embedding web content have been created, including the Swiss Parkinson App.

Patient education includes videos demonstrating exercises to be practiced by patients at their homes. The Park-in-Shape App was designed specifically for patients with PD and uses gamification to reward patients for completed exercises (van der Kolk et al. 2015). In this study, the active group showed 4.2 points higher scores in the off-state MDS-UPDRS as compared to the control. In addition, General exercise Apps can also be used by patients with PD.

Patient empowerment includes reminders for the (frequent) medication intakes. Many patients use simple alarm clock apps, but specific apps for medications have been developed (see Table 1 for examples). Many physicians would like to see patients acknowledge medication intakes to obtain information about patient adherence. Yet, many patients consider the use of apps as an extra burden. They feel they lack motivation, cite time constraints and feel that apps interfered with their established everyday-routines (Giebel et al. 2024; McBride et al. 2020; Post et al. 2024).

Symptom diaries have long been implemented for symptoms of PD; they require specific training and constitute a significant burden for patients, which can decrease compliance (Bendig et al. 2022). Still, obtaining more detailed information about the course and extent of symptoms can be critical to improve motor symptoms. Several digital symptom diaries are being developed, including the “ParkinsonHessen-Digital” project at University of Marburg.

Digital technologies may allow patients to monitor themselves and self-adjust their medication, similar to novel technologies about measuring blood glucose in diabetes mellitus. Awareness of symptom severity is impaired in patients with PD (Maier and Prigatano 2017). Technologies that monitor and report motor performance, fluctuations, or non-motor symptoms such as blood pressure may allow digitally-engaged patients to gain better control over their disease by learning to recognize factors that influence their symptoms.

Some PD healthcare apps include additional features such as diet. By being part of a larger platform, a “pocket dietitian” can offer personalized nutritional therapy and help users understand how diet influences their symptoms (e.g. NutriPD from My moves matter). Self-management systems for PD have also been recently reviewed elsewhere (Boege et al. 2024).

### Digital therapeutics

The classical digital therapeutic in Germany is the DIGA. Currently, no designated DIGA for Parkison patients exists, however, several other DIGA have been listed for symptoms that are present in PD patients, including depression, anxiety, pain, fatigue and others (Table 1). It is important to note that most of these DIGAs have not been specifically tested in PD patients. A good example is the DIGA NeuroNation that is certified for the application in cognitive impaired patients and requires the ICD-10: F06.7 to be registered for the individual patient. NeuroNation provides a multi-domain cognitive training that can be applied in patients with PD (Ferizaj et al. 2024; Maggio et al. 2022). So far, only feasibility data is available for PD. DIGA for patients with depression have recently been reviewed elsewhere (Haaf et al. 2024). Kranus Edera is a DIGA designed for men with erectile dysfunction. Elevida and untire are DIGA against fatigue, which demonstrated significant effects in patients with multiple sclerosis and cancer (Pöttgen et al. 2018; Spahrkäs et al. 2020). These DIGA can be applied in patients with PD if the respective ICD-10 code is registered for the patient. However, we recommend a careful observation of the benefit and usability of the DIGA since they have not been fully validated in PD patients.

Several related DHTs with therapeutic functional components that directly improve the patient’s situation have been developed (without DIGA-specific approval). If certified as medical device by the manufacturer, they can be applied in PD patients, but without a direct reimbursement by health insurances. One incapacitating, often medication-refractory PD symptom that has drawn considerable coverage by digital technologies is freezing of gait (FoG). Several apps offer various strategies to overcome or even prevent FoG episodes by recording the gait pattern which changes before FoG onset, and by addressing the main interictal gait disturbance characteristics predisposing to freezing episodes, as the loss of rhythm, the increased gait asymmetry and pathological gait variability. The apps offer auditory, visual, or tactile cues, to assist individuals in maintaining their rhythm and gait stability. A smartphone based DHT is the “Dual-Task Gait and Balance Assessment” app that enables the remote assessment of gait and balance in different real-world environments, including dual-task conditions by continuous characterization of the standing postural sway and gait variability (Su et al. 2021). It demonstrated significant correlations with gold-standard wearable motion sensors. The app “Rhythm – Parkinson’s Gait App” employs rhythmic auditory cues - or visual cues adapted by the smartphone camera - to enhance movement coordination. It detects walking patterns using on-device machine learning and plays rhythmic audio to support gait (Table 1). Another solution is the Apple watch- based app “Foggy” using the taptic engine of the Apple Watch to deliver vibrotactile stimulation when initiated by a simple patient´s tap, offering another non-invasive cueing strategy. For both Apps, efficacy data have not been published.

Medical devices to counter FoG include the “Holocue device”, a headset providing on-demand 2D and 3D holographic visual cues tailored to the user. This wearable application aims to alleviate FoG in real-world environments by offering external visual cues, but did not demonstrate immediate effects on FOG (Geerse et al. 2020, 2021). The “BIPBIP” is a wearable cueing system that integrates accelerometers to acquire data on gait and freezing, generating tactile stimuli when a freezing episode is imminent. The “Cue2Walk” is another wearable medical device which is attached to the lower limbs that simultaneously analyses step characteristics and starts vibratory or auditory cues when step length changes. For both Apps, efficacy data have not been published. Lastly, the app “CuPiD” provides repetitive training against FoG using exergaming with audio, visual and tactile feedback while patients train in different simulated environments. This training requires integration of cognitive and motor function in a motivating and engaging environment; feasibility data is available (Ginis et al. 2016).

## Telemonitoring and personalized interventions

Digital therapeutics – especially the German DIGA - are designed to be used by patients on their own. They need to provide a direct and independent benefit to the patient, and are generally used independently from healthcare professionals. Telemonitoring systems, in contrast, are based on the interaction between patients and healthcare professionals. They provide information on patients´ condition to the healthcare professional, facilitate care of patients in remote areas or facilitate direct communication (Fanciulli et al. 2025).

DHTs offer tools for personalized interventions that allow a shift from episodic, clinic-based evaluations to more continuous and responsive care models. Telemonitoring encompasses the continuous or periodic remote tracking of motor and non-motor symptoms. This involves a variety of technologies, including wearables like accelerometers and gyroscopes, smartphone-based applications, smart home sensors, and remote video assessments. These systems capture clinically relevant data quantifying impairments such as tremor, bradykinesia, dyskinesia, gait disturbances, falls, sleep patterns, and medication adherence from the typical everyday patient environment. One significant advantage of digital telemonitoring is its ability to provide healthcare professionals with objective health data with higher granularity than traditional visits. This can reveal subtle changes resulting from medication adjustments or disease progression, offering a more accurate picture of the patient’s day-to-day experiences. Digital technologies may be particularly valuable in patients with motor fluctuations in whom the motor state during clinic visits may not reflect real-world symptoms. Finally, digital technologies can record changes over longer timescales, which can be important for therapeutic effects (drugs and deep brain stimulation) on axial symptoms such as gait, which may become apparent more gradually. It is important to note, that continuous telemonitoring applications also might require immediate responses by healthcare professional in case of indicated emergency or related urgent treatment adjustments. Here, a good “digital literacy” and interplay between professionals and devices is needed to ensure continuous and responsive patient care.

Even though the DIGA as a digital therapeutic can include telemonitoring functionalities, the recipient of this monitoring information has to be primarily the patient. Thus, the telemonitoring solutions described above that interact between the patients and their healthcare professionals are rather complex digital supported healthcare solutions – referred to as “hybrid care” or “managed/digital supported integrated care” solutions, where technologies provided by their manufacturer have to work together with healthcare professionals and other services including medication, medical aids, and other tools. Thus, from a regulatory and reimbursement perspective, these hybrid telemonitoring systems are much more complex than DIGA, because they combine direct patient benefit (patient empowerment) with the clinical decision of healthcare professionals (clinical decision support) as well as an improvement of healthcare management efficiencies (care management support) such as managing medication change or appointments with other healthcare professionals or services. DIGA that support patient care, e.g. through self-management tools or symptom tracking, are considered “low-risk” medical apps** (typically risk class I or IIa under Medical Device Regulation) and must show a positive care effect to be prescribable. In contrast, telemonitoring systems that involve adjustments of treatment, such as change of medication or programming parameters, are classified as a medical device and have to prove safety and performance under MDR and have different requirements for reimbursement. Personalized interventions build upon sensor and other data by tailoring treatment approaches to each patient’s unique needs. Examples include fine-tuning dopaminergic therapy based on sensor data. For example, if a wearable device detects a motor off at 14:00, the treating neurologist might want to change the dosing of the 13:00 medication, or add a new drug like a COMT inhibitor. The benefits of treatment adjustments at higher granularity must be weighed against the additional burden to the patients if responses are required. Additional cost not only include the telemonitoring system itself, but also additional physician visits. Conceivably, this daunting task contributes to the fact that few telemonitoring systems have been implemented in routine care in Germany.

An illustrative example of an advanced telemonitoring system is ParkinsonGo by Portabiles, which pairs a shoe-worn sensor as a telemonitoring component, paired with an application that evaluates the patient´s self-perception (diary function), as well as tailored home-physiotherapy training videos, and guidance and support from a Parkinson nurse. Here, the combination of the monitoring function, with the movement intervention and the (human) support by a nurse aim to support the treating physician by continuous and telemedical care. This solution is reimbursed not by all healthcare insurances (yet) - but already received compensation within a selective care contract (this is a complementary reimbursement option in Germany for DHTs). A similar system is the PDMonitor Ecosystem by PD Neurotechnology. Both systems showed wearability and accuracy of measurements (Antonini et al. 2023), but efficacy of the telemonitoring concept has not been published yet. Other examples include implementing adaptive deep brain stimulation driven by telemetric input, and creating individualized physical activity plans through remote coaching. Study protocols and feasibility have been published (Schootemeijer et al. 2023; van den Bergh et al. 2023).

Impulse control disorders (ICD) are a significant non-motor complication in Parkinson’s disease, often linked to dopaminergic therapy (Witt et al. 2024). Digital technologies show promise for early ICD detection and monitoring through wearable EEG-based cognitive tasks (Lin et al. 2021). Additionally, digital delivery of cognitive-behavioral interventions offers a scalable approach to managing these behaviors (Mena-Moreno et al. 2022; Okai et al. 2013). Hence, integrated digital solutions to detect and treat ICD can be envisioned for the future. In the future, AI-driven predictive analytics, expanded monitoring of non-motor symptoms, and hybrid models that blend digital monitoring with traditional care frameworks are envisaged.

## Patient management tools along the individual´s journey

Digital technologies can also support the management of patients along their individual patient journey. When requiring numerous different healthcare professionals, patients usually depend on their own management skills and their informal caregivers. In this context, is often challenging for the individual patient to identify physicians and therapists with experience on PD. Several online portals already exist. The project “Parkinson-Lotse”, for instance, is a web portal that provides a map search to help patients find qualified providers with a focus on non-drug therapies. Selection criteria for healthcare providers to be listed on the map have been developed with PD experts of the association “Parkinson-Netzwerke Deutschland e.V”, thus constituting a quality assurance system that sets this web portal apart from others. Another platform, “JamesAKTIV” is currently tested in the project ParkinsonAKTIV to support interdisciplinary exchange between physicians and non-medication therapists. For instance, neurologists may enter specific therapeutic targets into the portal whenever they prescribe physiotherapy. This information is seen by the physiotherapists treating the patient, who may modify the treatment strategy based on their assessment and provide feedback to the prescribing physician (Achtert et al. 2023). Digital technologies are also heavily used to manage patients’ appointments, but this area is not covered further here. However, with growing health-data exchange platforms such as the national electronic health record and related capabilities of DHTs to even include AI-assisted management support solutions, this aspect will become relevant very soon – especially due to their efficiency, availability, and the fact, that the majority of patients consult electronic information platforms before they visit the doctor.

## Endpoints for clinical trials

Digital technologies – especially digital diagnostics - comprise several promises with respect to clinical trials. By objectively and remotely measuring clinical signs repeatedly, digital technologies can, in principle, monitor progression or fluctuations in symptom severity across time ranging from minutes to months. Fluctuation of motor and non-motor symptoms is common for patients with PD, but has also been described for patients with cerebellar diseases where it has not been widely appreciated before (Grobe-Einsler et al. 2021). Moreover, symptoms might be captured with more granularity by digital technologies than, for instance, by a clinical rating scale where typically a score of 0, 1, 2, or 3 points is assigned to each symptom. Both properties potentially lower the number of participants required to show significant effects of symptomatic and disease modifying therapies, which would significantly accelerate drug discovery. Digital technologies also allow assessment of symptoms in patients’ homes and - potentially - assessment of symptom impact on patients’ lives, which will be important for regulatory approval in the future. Moreover, remote study visits can reduce the logistical burden for patients in clinical trials and help to contain costs. In this context, digital technologies can record activities other than those affected by dopaminergic mediations, potentially facilitating the study of drugs that modify the progression of the disease. Remote study visits can be complemented by patient reported outcome or experience measures (PROMs/PREMs), motor assessments, but also assessments of non-motor symptoms such as blood pressure fluctuations.

Examples of effective digital assessments for PD motor symptoms include reporting of tremor by smartwatches, assessment of gait using wearable sensors and video acquisition (Adams et al., 2024; Cohen et al. 2018; Moreau et al. 2023; Powers et al. 2021; Raschka et al. 2025). Voice recordings still require specialized equipment for high quality analysis, but can provide quantitative readouts of hypophonia and dysarthria in patients with PD (Hähnel et al. 2025; Thies et al. 2025; Tröger et al. 2024). Digital technologies have been explored as readouts for cognitive function, for instance using chatbots to record verbal fluency tasks, by in depth analysis of verbal fluency lists, and using digital tests adapted from psychophysical experiments (Berron et al. 2024; König et al. 2024). Finally, continuous monitoring of blood pressure may be applied for monitoring hypotensive episodes in people with PD - both in studies for treating neurogenic orthostatic hypotension and for monitoring the effect of therapeutic changes that potentially impact on blood pressure such as 24 h-infusional therapies. Cuffless technologies are increasingly available, which may minimize patient burden. Reliability in case of severe, position-dependent blood pressure fluctuations remains, however, to be ascertained (Derendinger et al. 2024; Moharram et al. 2020; Socrates et al. 2021).

## Discussion

In this narrative review, we describe areas of development for DHTs and other digital resources in the context of Parkinson’s disease. Overall, developments appear to be driven primarily by novel technical innovations, and are followed by new regulatory/assessment frameworks relevant for evidence-based medicine and reimbursement of beneficial DMDs. For patient education and empowerment, these possibilities include the availability of web portals to host educational material and the possibility to facilitate access to these repositories through smartphone apps. Assessment of motor symptoms is based on the availability of inertial sensors in wearable consumer devices, notably smartwatches, and the development of analysis techniques for these complex signals. In addition, smartphone cameras and analysis possibilities embedded into the smartphone operating system offer novel possibilities for home monitoring and telemedicine. From a healthcare practitioner perspective, the possibility to provide insight into symptom severity and impact beyond the half hour they typically spend in our offices constitutes the most important paradigmatic change offered by digital technologies.

Seeing these resources raises the question why not more PD-specific Apps have been created. One possible explanation could be that many patients with PD prefer general Apps for lack of stigma (Bendig et al. 2022). A second explanation could be that general Apps are not regulated tightly whereas it would be hard for a PD-specific App to avoid claims of being a DIGA or medical device, which entails significant regulatory burden. An important hurdle comes from the low “digital literacy” of healthcare professionals. Here, training programs included in academic and non-academic education curricula, as well as continuous education programs are needed. Healthcare professionals lack an understanding of how to use DHTs themselves – e.g. in hybrid/telemonitoring applications, how to recommend DHTs to their patients – e.g. explaining the functionality of DIGAs, and how to guide on good and validated DHTs – e.g. as part of the professional guidelines. For the manufacturer of DHTs, programming/manufacturing medical devices requires extensive technical documentation and tight quality management processes for regulatory approval and registration in the European database on medical devices (EUDAMED). In addition, medical devices need to be tested in clinical trials before they can be distributed – and the methodological experience in clinical researchers to evaluate e.g. patient-centered outcomes such as health literacy, autonomy, self-efficacy is yet limited. The clinical research expertise and capabilities are also required for post-market surveillance studies – a requirement similar for drugs.

Also, usability aspects are a hurdle for successful adoption of digital tools by patients: While interacting with DHTs, people with PD may encounter difficulties, potentially due to their motor or cognitive impairment, or rather limited digital literacy of older generations. These constraints need to be taken into account when designing new digital devices and policies. In addition, patient education is crucial for their successful implementation. This education needs to inform patients about their disease (health literacy) and about the functioning of digital technologies (digital literacy). Digital literacy is also required for healthcare professionals, and all partners require continual technical support. Novel digital technologies need to be integrated with existing software in hospitals and offices, and measures obtained by different technology providers need to be aligned. For telemonitoring systems in particular, the resources required for improved symptom control (digital devices, network services, nurses, physicians) need to be weighed against the functional benefits for patients’ everyday lives. Finally, cybersecurity and reimbursement aspects need to be considered.

To some extent, it would be desired to base future developments more on needs voiced by patients and/or healthcare practitioners – an increasing research field referred to as public-patient-involvement (PPI) or responsible research innovation (RRI). This aspect is of particular relevance for DHTs since – in contrast to drugs – the usability requires a much broader attention than swallowing a prescribed pill. The mixed response of patients to symptom diaries and medication reminders illustrate the point that interests of patients and healthcare professionals are not always aligned. In this respect, the regulatory distinction between DIGA and other medical devices offers guidance to decide - already during development - whether a product is primarily intended for patient empowerment (e.g. DIGA) or to support healthcare professionals as telemonitoring solutions, or both, as hybrid or complex telemedical platforms and services. The technologies in these application areas have to fulfill regulatory assessment requirements that are currently also changing on national and international levels.

The opposition by some patients and healthcare practitioners against digital technologies may relate to the idea of digital technologies leading to a “healthcare machine”. This concern is not without reason, in particular given the reduced capacities in the healthcare system. At the same time, however, digital technologies already are providing manifold opportunities for patients to take a more active role in managing their disease and for allowing more individualized treatment regimens. It will be interesting to see whether computer-generated treatment suggestions have higher or lower effects than current standard of care. Finally, digital technologies can reach large audiences and thereby may facilitate achievement of the Brain Health Mission, i.e., to reduce the global burden of neurological diseases, by offering supporting exercise, controlling risk factors and preventing social isolation.
